# Recent advances in microbial fermentation for dairy and health

**DOI:** 10.12688/f1000research.10896.1

**Published:** 2017-05-26

**Authors:** Daragh Hill, Ivan Sugrue, Elke Arendt, Colin Hill, Catherine Stanton, R Paul Ross

**Affiliations:** 1 Department of Biosciences, Teagasc Food Research Centre, Moorepark, Fermoy, Cork, Ireland; 2 APC Microbiome Institute, University College Cork, Cork, Ireland; 3 The School of Food and Nutritional Sciences, University College Cork, Cork, Ireland; 4 School of Microbiology, University College Cork, Cork, Ireland; 5 College of Science Engineering and Food Science, University College Cork, Cork, Ireland

**Keywords:** microbial fermentation, prebiotic, fermented diary product

## Abstract

Microbial fermentation has been used historically for the preservation of foods, the health benefits of which have since come to light. Early dairy fermentations depended on the spontaneous activity of the indigenous microbiota of the milk. Modern fermentations rely on defined starter cultures with desirable characteristics to ensure consistency and commercial viability. The selection of defined starters depends on specific phenotypes that benefit the product by guaranteeing shelf life and ensuring safety, texture, and flavour. Lactic acid bacteria can produce a number of bioactive metabolites during fermentation, such as bacteriocins, biogenic amines, exopolysaccharides, and proteolytically released peptides, among others. Prebiotics are added to food fermentations to improve the performance of probiotics. It has also been found that prebiotics fermented in the gut can have benefits that go beyond helping probiotic growth. Studies are now looking at how the fermentation of prebiotics such as fructo-oligosaccharides can help in the prevention of diseases such as osteoporosis, obesity, and colorectal cancer. The potential to prevent or even treat disease through the fermentation of food is a medically and commercially attractive goal and is showing increasing promise. However, the stringent regulation of probiotics is beginning to detrimentally affect the field and limit their application.

## Introduction

The fermentation of food by microbes has been employed for millennia as a process to ensure extended shelf life and improve the functionality, texture, and flavour of food products. The first evidence of dairy fermentation exists from approximately 7,000 years ago, where early Europeans are thought to have produced cheese ^1^. Methods have evolved from spontaneous fermentation by the indigenous microbial population to pre-selection of starter cultures with known attributes. Lactic acid bacteria (LAB) are the major bacteria used in food fermentations worldwide. LAB consist of a myriad of genera including, but not limited to, *Lactobacillus, Lactococcus, Streptococcus, Leuconostoc, Pediococcus*, and *Enterococcus*. Though the LAB are a diverse group of bacteria, many species enjoy historical “generally regarded as safe” (GRAS) and “qualified presumption of safety” (QPS) status by the Food and Drug Administration (FDA) and European Food Safety Authority (EFSA), respectively ^2^. LAB fermentation has long been recognised to confer beneficial effects on human health through the modulation of the intestinal microbiota. These either directly or indirectly affect the host microbiota, which in turn can lead to an effect on health. The use of these bacteria in fermentations to produce functional foods has greatly increased in recent years. Consumption of fermented foods has been associated with a range of health benefits from disease prevention to enhancing the bioregulation of behavioural issues such as stress and anxiety ^3– 5^.

**Figure 1.**
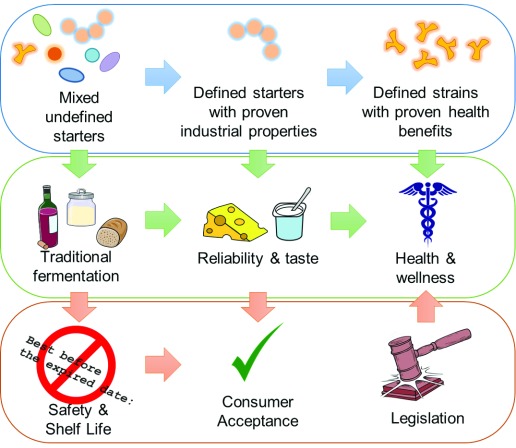
Schematic representing the relationships among fermenting microbes, fermented dairy products, and the consumer.

While the consumption of traditional fermented foods in cultures around the world is believed to have beneficial effects, not all of these foods have been subjected to appropriate trials in which these beliefs could be credited or discredited. The benefits of fermented dairy products are being researched extensively in parts of the world, but other ethnic fermentations are also beginning to be studied in more detail ^6^. These traditionally fermented foods use uncharacterised starter cultures that could possess novel properties or be useful in other fermentations, some of which will be discussed in more detail later ^6– 8^. The potential application of microbial fermentation is enormous both in health and in biotechnology and will be an important area of research and production in the coming decades ^9^. Yeasts and moulds are also prominent fermenting organisms in alcoholic and certain cheese fermentations. The focus of this review is to look at advances in the past three years in the field of microbial fermentation with a focus on food and added health benefits of fermentation, including extraneous commercial and legislative factors impacting the field ( Figure 1).

## Fermentation starter cultures and by-products of fermentation

**Figure 2.**
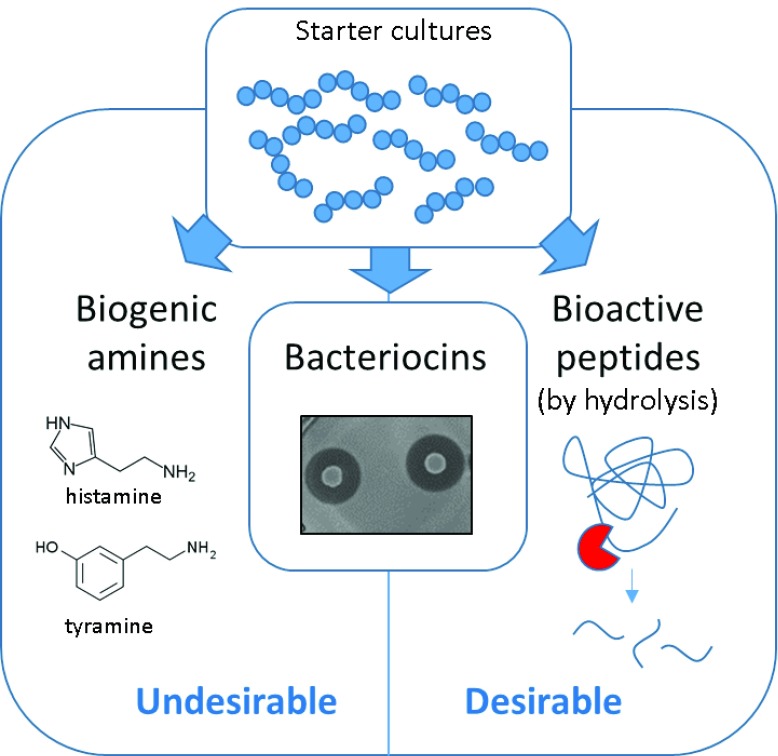
Desirable and undesirable bioactive metabolites produced during fermentation which can impact choice of starter cultures. Fermentation starters can produce a number of desirable and undesirable bioactive metabolites. Biogenic amines (left) are an undesirable product in most fermentations due to their toxicity. Bioactive peptides (right) produced through enzymatic release are desirable by-products due to positive biological activity. Bacteriocins (centre) are desirable as a known probiotic trait, but potentially undesirable in a starter culture due to possible impact on other fermenting cultures.

Starter cultures, which carry out the fermentation process, are used to ensure consistency in commercial products by using known species with desirable traits, such as a high rate of acidification through the production of lactic acid and/or the secretion of secondary metabolites into the fermentate matrix ( Figure 2). Novel starter cultures are continually in demand for the development of new commercial products along with greater characterisation of those currently in use to ensure safe and functional products. There are many positive and negative factors that impact the selection of starter cultures in dairy fermentations, such as a history of safe use, acidification rate during fermentation, exopolysaccharide production ^10^, proteolytic activity, particularly during cheese production, and the generation of bioactive metabolites and peptides ^11, 12^.

## Bacteriocins

Bacteriocins are small ribosomally synthesised antimicrobial peptides against which the producer species is immune and which act against other bacteria in a bactericidal or bacteriostatic manner ^13^. Great care must be taken with regard to bacteriocin production in starter cultures, as they may target other fermenting cultures or adjuncts; however, their ability to inhibit potential spoilage bacteria and pathogens can be of great use ^14^. The identification of bacteriocinogenic strains has mostly relied on agar diffusion-based assays ^15^. Increasing interest in bacteriocins as alternatives to antibiotics and chemical food preservatives has led to new methods for identifying bacteriocin producers. *In silico* screening using programs such as BAGEL ^16^ and antiSMASH ^17^ enables the discovery of new bacteriocin operons where whole genome data are available. Such methods avoid any potential problems with unsusceptible indicator strains ^18^ and can allow for faster initial screening. Collins *et al.* (submitted, 2017) using an *in silico* screen of the genomes of 213 lactobacilli ^19^ identified 11 bacteriocins, five of which were novel. Another *in silico* study which mined human gut microbiome sequence data found 74 bacteriocin gene clusters from 382 fully sequenced genomes ^20^. *In silico* screens such as these rely on previously described peptides or the identification of bacteriocin accessory genes, and as such it is unlikely that initial agar diffusion-based assays will be completely replaced with *in silico* screening. However, they may represent an opportunity to search for new bacteriocins in complex microbiotas such as those of a traditional fermented product.

## Biogenic amines

Biogenic amines (BAs) are biologically active, low-molecular-weight organic bases produced mainly through the decarboxylation of certain amino acids, which can accumulate during fermentation. Traditionally, the presence of BAs in food products is associated with undesirable microbial activity, indicating food spoilage or defective manufacture ^21^. Dairy products, in particular cheese, can accumulate high levels of BAs, mainly histamine and tyramine, which are known to be toxic ^22– 24^, but as of yet legal limits have been set only for histamine in fish products ^25^. The accumulation of more than one kind of BA in products is of particular concern owing to their synergistic toxicity at dietary concentrations, which has recently been demonstrated with intestinal cells *in vitro* ^26^. BAs are detected in dairy products by the chromatographic detection of BA compounds or through the detection of BA-producer organisms using PCR-based methods, which correlate with HPLC results ^27^. In a recent study, levels of tyramine in a model cheese were reduced by 85% through the use of a bacteriophage to limit the population of BA-producing bacteria ^28^. Pre-selection of starter cultures lacking BA genes in the future may be necessary to avoid unwanted build-up of BA compounds and continued avoidance of contamination, which is known to occur during post-ripening processing ^29, 30^.

## Bioactive peptides

Bioactive peptides are encrypted in larger proteins and, when released after proteolysis, have been associated with health promotion through a number of mechanisms such as inhibition of angiotensin 1-converting enzyme (ACE) activity, antithrombotic activity, antihypertensive activity, antioxidant activity, immunomodulation, apoptosis modulation, and by opioid and anti-opioid activities ^11, 31^. LAB possess a myriad of proteases and peptidases that can release encrypted peptides during milk fermentation ^32^ or following the ingestion of fermented products containing LAB in the intestinal lumen. In recent years, potential anticarcinogenic peptides have been found encrypted in bovine milk casein and whey proteins, including the previously known cationic lactoferricin ^33^. The known cancer-preventative peptide lunasin has been found to be proteolytically released during sourdough fermentation by LAB ^34^. Further research has subsequently revealed increased protease resistance during *in vitro* gastrointestinal transit in the presence of naturally occurring protease inhibitors to allow lunasin to reach the large intestine ^35^. Another recent study has found that the administration of milk fermented by a probiotic *Lactobacillus casei* strain modulated the immune response against a breast cancer tumour in a mouse model, with delayed or blocked tumour development in the fermented-milk-fed group as compared with unfermented milk as a control ^36^. The mechanism of action has not yet been elucidated, and studies of a similar nature have not yet passed the animal trial preclinical stages of investigation.

## Ethnic fermented milk products

There is increasing interest in novel LAB strains isolated from ethnic fermented milk products, which would have been part of the autochthonous fermenting microbiota. Products such as matsoni, a fermented milk product of Armenian origin, and kule naoto, the traditional fermented milk product of the Maasai in Kenya, are having their previously undescribed microbiotas characterised using sequencing-based analysis ^37, 38^. Indeed, such products may be of great value: for example shubat, a probiotic fermented camel milk of Kazakh origin, has recently been found to demonstrate positive hypoglycaemic activity in type 2 diabetic rats ^39^, and the indigenous Indian fermented beverage Raabadi has been investigated as a source of probiotic hypocholesterolaemic lactobacilli ^40^. However, a study of mursik, a milk product from Kenya that is traditionally fermented in a gourd, has been suggested as a possible etiological factor for oesophageal cancer because of high levels of ethanol and acetaldehyde present post fermentation ^41^. This is in stark contrast to the health claims made for most commercial fermented dairy products that indicate the benefits of using safe, known starter cultures.

## Prebiotics

In 2008, prebiotics were defined by the International Scientific Association for Probiotics and Prebiotics (ISAPP) as “a selectively fermented ingredient that results in specific changes in the composition and/or activity of the gastrointestinal microbiota, thus conferring benefit(s) upon host health’’ ^42^, a definition that is currently being further revised by ISAPP. Prebiotics are fermented by the gastrointestinal microbiota and contribute to healthy modulation of the gut ^43^. The ingestion of specific prebiotics has been shown to increase antibacterial capabilities of a probiotic strain ^44^. Synbiotics are a relatively new area that involve a combination of probiotic and prebiotic in one product; the prebiotic is intended to improve the survival/growth/performance of the probiotic or other beneficial bacteria in the colon, which in turn has beneficial health effects on the host ^45^.

Currently, research is being conducted on the role of the gut microbiota in the development of cancer, with a focus on colorectal cancer ^46^. While this research is in its early stage, there is evidence for the use of probiotics, prebiotics, and synbiotics in the treatment or prevention of this disease. There is potential for these to act as anticarcinogens or antimutagenic agents through diet-based interventions. More detailed analysis could lead to huge strides in the prevention of cancer, but as of yet the field is open to new research ^47^.

## Health-focused research

Research into the use of fermented foods as a potential approach to fight disease is growing, but it must be appreciated that many of these functional foods are intended to prevent disease onset, or alleviate symptoms, and not necessarily act as a curative agent ^48^. This increases the burden of proof on the researcher to prove that the fermentation of the prebiotic was indeed the reason the host remained healthy. Modulation of the gut microbiota is the focus of many studies relating microbial fermentation to measureable health benefits. One emerging area of study using microbial fermentation is in osteoporosis. Osteoporosis is common in postmenopausal women and the elderly and presents itself as weakened bones prone to breaks or fractures due to poor calcium absorption ^4^. The consumption of a prebiotic fructo-oligosaccharide (FOS) has the potential to be a preventative method for osteoporosis ^49^. The prebiotic is fermented in the gut, causing a drop in the pH of the lumen to such an extent that previously insoluble calcium phosphate will dissolve. This plays a beneficial role in bone mineral density ^50^. The fermentation of FOS releases short-chain fatty acids and lactic acid, which cause the drop in pH. This dissolved calcium results in an increase in passive diffusion; thus, it could help to treat, or even potentially prevent, the onset of osteoporosis.

Obesity is a global issue and has generated much interest in whether and how our gut bacteria could be a contributing factor in the development of this complex syndrome. On-going studies into the gut microbiota are aimed at identifying whether a specific bacterium or bacterial group could be contributing to obesity ^51^. While this is an emerging area of research, there are exciting developments on how to potentially fight this syndrome through the modulation of the gut microbiota ^52^. The so-called “obese microbiota profile” can be characterised as a decreased Bacteroidetes/Firmicutes ratio in individuals ^51^. One study looked at the administration of prebiotics such as FOS as a potential method of reducing the likelihood of obesity by increasing the levels of “lean microbiota” through fermentation of the prebiotic in the gut. This in turn led to a decrease in the permeability of the intestine with improved tight junction integrity. While this is in early stages of research, it does present a potentially new method by which obesity could be treated through microbial fermentation within the gut ^53^. This area of research has great potential medically and commercially ^54^.

Bacteriocins, as discussed previously, are antimicrobial peptides that target and kill other bacteria and that could potentially be utilised as an anti-obesity tool. If specific strains that are contributing to obesity are identified, then modulation of the gut microbiota, through introduction of specific bacteriocins or bacteriocin producers has the potential to reduce the risk of obesity. Likewise, specific bacteriophages could be used to target such strains.

Current evidence supports the notion that microbes in the gut could be a contributing factor to mental disorders through the brain–gut axis ^55^. Psychobiotics are an emerging area of study on the role of the microbiota in brain health. A psychobiotic is a bacteria that, when ingested in adequate amounts, can have a positive mental health benefit ^56^. The permeability of the intestinal barrier can be compromised by the westernised diet of processed foods and carbonated beverages. The bacteria present in our gut are capable of producing neurotransmitters through the metabolism of indigestible fibres; these include dopamine, noradrenalin, GABA, and acetylcholine ^57^. The consumption of probiotics in fermented foods could have a positive influence on maintaining the intestinal barrier and preventing chronic inflammation. Dietary interventions in adolescents of more fermented foods containing beneficial brain bacteria could help prevent the onset of depression and anxiety, among other mental health issues, which are becoming more prevalent.

## National recommendations

Fermented foods have been consumed worldwide for thousands of years before any direct health benefits were truly understood. While the demand from consumers for functional foods is growing, the national recommendations are not following suit. Now that the mechanisms by which these fermentations can beneficially affect human health are beginning to be elucidated, food guidelines around the world are slowly beginning to recommend their consumption. This inclusion is not universal, despite historical use and clinical trials proving the benefits of these fermented products in the diet. Given the strong tradition of fermented foods in Asia, it is somewhat surprising that they are not specifically included in food guidelines, albeit the Chinese Nutrition Society suggests the consumption of yogurt for those who do not tolerate lactose well.

There is a high incidence of lactose intolerance in Asian countries, and there are clinically proven studies that show the inclusion of fermented dairy foods can help to alleviate the symptoms of intolerance ^58^. Japanese authorities list fermented foods in the Food of Specified Health Use (FOSHU) category, and in India the guidelines specifically encourage the consumption of fermented foods. The Indian guide highlights specifically that pregnant women should consider including more fermented foods in their diet owing to the increased bioavailability of iron that is associated with these foods.

## Regulation of fermented dairy products

The regulation surrounding microbial fermentations in the food industry is beginning to have a detrimental effect on the industry as a whole. For example, there is currently no legal definition for the term “probiotics”; until scientific, legal, and industrial teams are all working together under one solid definition, the term “probiotics” will begin to lose its meaning. Along with this, the general community are losing confidence in the benefits of fermented dairy products that are supplemented with probiotic/prebiotics. Since December 2012, in Europe, labelling of a probiotic was banned along with the use of health claims in any product without receiving approval from EFSA, which has yet to approve any probiotic health claim. This is despite the numerous clinical trials proving the benefits of probiotic yogurts in health. This change has led to consumer confusion as to whether or not the claims were ever true. It is essential that labelling of fermented food products with clinically proven health benefits is permitted to allow industry to begin to profit from funding these trials, or they will begin to invest in marketing strategies rather than the much-needed research ^42^. The International Dairy Federation (IDF) represents the dairy sector at relevant codex meetings regarding the international standards for dairy products ^59^. The IDF are currently involved in investigating product labelling with regard to nutritional information and health claims and how these affect the consumer’s choice of different products ^60^. These studies will hopefully lead to a change in labelling laws to allow for clinically proven health claims to be present on fermented dairy products.

## Conclusion

Microbial fermentation holds the key to some extremely complex interactions between bacterial species and the food matrix they are fermenting. The studies highlighted in this review show the potential of utilising these microbial fermentations in a more knowledge-based fashion than that of the past. With regard to microbial fermentation in food, this represents an area with potential well beyond the extension of shelf life. The work in these areas is continuing and, with the help of better regulation, could lead to exciting new discoveries on managing disease symptoms through food. Though fermented products have long been associated with health promotion, the lack of regulation has been a confounding factor in consumer attitudes. Indeed, other legislation must be put in place in the near future for harmful levels of BAs in fermented dairy products, given that at present no upper limit for potentially toxic levels of histamine and tyramine are available. The search for probiotics is on-going using both genetic and traditional screening methods ^61, 62^. Probiotics have a bright future in the area of supplemented fermented foods for health promotion.

There have been numerous advances in fermented products, the microbes which produce them, and fermentable polysaccharides in recent years. With public opinion shifting towards healthier lifestyles and viewing chemical preservatives in a negative light, fermented products show great commercial promise. New starter cultures are being identified using more sophisticated methods to ensure their effectiveness and viability. *In silico*-based methods and research in the health-promoting activities of LAB in fermentates are on the rise, along with the characterisation of traditional products that have been associated with good health.
